# Editorial: Innovative Technologies and Clinical Applications for Invasive and Non-invasive Neuromodulation: From the Workbench to the Bedside

**DOI:** 10.3389/fneur.2019.01350

**Published:** 2020-01-10

**Authors:** Matteo Bologna, Aristide Merola, Lucia Ricciardi

**Affiliations:** ^1^Department of Human Neurosciences, Sapienza University of Rome, Rome, Italy; ^2^IRCCS Neuromed, Pozzilli, Italy; ^3^Department of Neurology, Wexner Medical Center, The Ohio State University, Columbus, OH, United States; ^4^Neurosciences Research Centre, Molecular and Clinical Sciences Research Institute, St. George's University of London, London, United Kingdom

**Keywords:** deep brain stimulation (DBS), Non-invasive focal mechanical vibrations (NIFMV), Parkinson's disease (PD), spinal cord stimulation (SCS), transcranial magnetic stimulation (TMS), transcranial ultrasound stimulation (tUS)

Invasive and non-invasive brain stimulation represent one of the most promising scientific advances of the last decades (1). This special issue was designed to highlight and critically discuss innovative neurostimulation procedures for Parkinson's disease (PD) and other neurological conditions. Of the 17 papers initially submitted to the journal by international researchers, 13 were considered suitable for publication after a thorough peer-review process. These included five original researches, five reviews, one systematic review, one brief research report, and one case report. The following is a short summary of the main results of each of these manuscripts.

Deep Brain Stimulation (DBS) of the subthalamic nucleus (STN), the ventral-intermediate nucleus (VIM), and the globus pallidus pars interna (GPi) are well-established therapeutic options for medically refractory PD, essential tremor, and dystonia (2, 3). However, several aspects related to DBS programming and post-surgical management of medications still remain to be clarified. In their manuscript, Koeglsperger et al. provide a concise review of strategies for DBS programming and dopaminergic medications adjustments following DBS. In another review from the same group, by Hell et al., summarizes and carefully discusses future perspectives for DBS, including target identification, adaptive closed-loop stimulation, and associated feedback signals.

Although STN- and GPi-DBS are both considered effective in reducing levodopa-induced dyskinesia (LID), the comparative efficacy of the two targets on dyskinesia remains unclear. Liu et al. conducted a meta-analysis of studies reporting data on STN- and GPi-DBS efficacy on LID. The authors found that GPi-DBS may reduce dyskinesia to a higher extent than STN-DBS at 12 months. This observation implies that mechanisms for dyskinesia reduction may be different between STN- and GPi-DBS. Future studies are needed to clarify the complex biological interaction with different systems of fibers involved in the modulation of motor symptoms in the two most common targets for DBS in the basal ganglia.

STN-DBS may also have a beneficial effect on balance and gait in PD. However, published results yielded variable conclusions. In their prospective controlled study, Szlufik et al. evaluated the impact of STN-DBS on balance disorders in PD. The authors found a beneficial effect of STN-DBS on static and dynamic instability in the short-term follow-up, while long-term data remain controversial. Zhang et al., report the case of a PD patient implanted with STN-DBS and complaining of severe speech problems. Tremor and speech problems were both effectively treated by a novel stimulation procedure, i.e., variable-frequency stimulation (VFS) consisting of a combination of high frequencies. This preliminary observation should be confirmed in future controlled studies. Again, concerning the possible detrimental effects of DBS, the review paper titled: “A Review of Cognitive Outcomes Across Movement Disorder Patients Undergoing Deep Brain Stimulation,” by Cernera et al. discuss the issue of DBS-associated cognitive declines and adverse effects on quality of life in PD and other movement disorders. Pathophysiological mechanisms for cognitive changes occurring after DBS are also discussed.

Non-invasive neuromodulation, including Transcranial Magnetic Stimulation (TMS), in movement disorders is a challenging issue for both clinical and research purposes (4–6). Various non-invasive brain stimulation protocols have been studied in different conditions and settings. Hence, the reliability and validity of non-invasive neuromodulation techniques are still to be elucidated. In this regard, various methodological factors, possibly influencing the outcome measure, need to be better investigated. Fricke et al. developed an associative dual-site rTMS (1 Hz) targeting the premotor and primary motor cortex. The protocol aimed to activate different cortico-basal ganglia projections and, therefore, to target pathogenic oscillations at distinct STN subregions. The study results demonstrate that the stimulation was tolerated well, but did not improve motor symptoms in PD. Even though results were negative, this study raises interest toward non-invasive treatment options for PD symptoms. Furthermore, negative therapeutic results should not discourage the use of non-invasive stimulation techniques as a tool to investigate pathophysiological mechanisms underlying movement disorders. For example, dystonia is a relatively frequent movement disorder with unclear pathophysiology. However, the cerebellum is now considered a key area in the generation of dystonic symptoms. Odorfer et al. combined non-invasive cerebellar stimulation, i.e., continuous theta-burst stimulation and functional magnetic resonance imaging techniques, and investigated simple finger tapping in patients with cervical dystonia. Results indicate that finger movements, although clinically normal, are associated with altered cerebellar activity, further supporting the hypothesis of a prominent cerebellar involvement in dystonia. Finally, it should be considered that non-invasive stimulation techniques also allow to investigate physiological aspects not necessarily involved in the motor control. For example, mesial cortical areas in the frontal lobe and the ventral striatum are key nodes involved in decision-making and executive functions. In their original research study, including neuroimaging techniques, Popa et al. demonstrate how deep inhibitory rTMS can influence the underlying network functional connectivity of the targeted mesio-prefrontal-cingulo-striatal circuits regions. The study emphasizes that the modulation of resting neural activity in mesial prefrontal-striatal circuits by non-invasive techniques as a potential therapeutic tool for a wide range of psychiatric and neurologic disorders, particularly drug-cue reactivity processes relevant to addiction.

An increasing number of studies on animals and humans suggest that both the peripheral and the central nervous system can be targeted and potentially modulated by ultrasound stimulation techniques (7). An important aspect concerns the possibility to suppress or facilitate ongoing neural activity (during stimulation), as well as to induce long-lasting effects or even tissue ablation. In their review paper, di Biase et al. summarize mechanisms of actions, stimulation parameters, and therapeutic application of Transcranial Ultrasound Stimulation (tUS) in healthy humans and various disease states. In their original research article, Gibson et al. demonstrate that tUS delivered via a commercially available diagnostic imaging ultrasound system transiently increases excitability in the motor cortex. The results raise the intriguing possibility of new clinical applications for this technology, mainly as a diagnostic imaging for neuroplasticity induction not only in the motor cortex, but also in other brain areas. This initial but promising results encourage further research studies.

Alternative non-invasive stimulation techniques, like Spinal Cord Stimulation (SCS) and Non-Invasive Focal Mechanical Vibrations (NIFMV), may also improve motor control in different neurological diseases (8, 9). The review paper by Fonoff et al. summarizes the most relevant advances from experimental and clinical studies, including anecdotal reports, on SCS for gait disorders. The author discusses the potential mechanisms of action, neural substrates, and clinical outcomes of SCS, suggesting that gait abnormalities in parkinsonian syndromes, particularly freezing of gait, can improve with SCS. However, the authors acknowledge that future well-designed trials are needed to delineate the possible therapeutic applications for SCS. The results of the pilot open-label trial by Schirinzi et al. indicate that NIFMV represents a feasible, safe, and effective option of supportive therapy for patients with cerebellar ataxia. More extensive controlled studies are necessary to confirm these preliminary observations and to define other critical methodological aspects related to treatment and eligibility criteria.

In conclusion, the editors wish to thank all the authors, the reviewers, and the editorial board members for contributing to this special issue. We hope that this special issue might inspire future and novel research approaches in the field of invasive and non-invasive neuromodulation in Parkinson's disease and other movement disorders.

## Author Contributions

All authors listed have made a substantial, direct and intellectual contribution to the work, and approved it for publication.

### Conflict of Interest

The authors declare that the research was conducted in the absence of any commercial or financial relationships that could be construed as a potential conflict of interest.

## Abbreviations

DBS: Deep Brain Stimulation
GPi: Globus Pallidus internus
LID: levodopa-induced dyskinesia
NIFMV: Non-Invasive Focal Mechanical Vibrations
PD: Parkinson's disease
SCS: Spinal Cord Stimulation
STN: Subthalamic Nucleus
TMS: Transcranial Magnetic Stimulation
tUS: Transcranial Ultrasound Stimulation.
